# The Ministry and Unregistered Dentists

**Published:** 1920-03-27

**Authors:** 


### THE MINISTRY AND UNREGISTERED DENTISTS.

At the annual meeting of the National Dental Association, recently held at the Connaught Rooms, London, the following protest to the Ministry of Health was unanimously adopted:

"Resolved that this annual meeting of the National Dental Association strongly protests against the inactivity of the Ministry of Health in not having introduced (eleven months after the date of the report of the Departmental Committee on Dentistry) legislation which would have the effects of automatically eliminating the advertising and dental quack. And it is resolved further that strong representations be made to the Ministry that, not only in the interests of both sections of the dental profession but also in those of the public health, some measure should be at once introduced into the House of Commons dealing with dental matters in order adequately to protect the public from the gross evils referred to in the Departmental Committee's report, which was signed by representatives of the medical profession, both sections of the dental profession, and laymen."

This resolution was duly sent to Dr. Addison by Mr. Horace P. Bastow (the general secretary of the organisation), and the following reply came to hand:

*Re* Dental Bill.

"I am directed by the Minister of Health to acknowledge the receipt of your letter of the 4th inst., forwarding a copy of a resolution passed by the National Dental Association regarding the recommendations of the Departmental Committee on the Dentists Act and in reply to state that it is hoped *to introduce legislation on the subject during the present session.*"

It is interesting to find that the characteristic lethargy of Government departments does not afflict the Prime Minister or the Minister of Health, who are both apparently keenly alive to the dental needs of the population of Great Britain.

Also we have received a copy of a letter from the pen of J. P. Blizard, Esq., J.P., who is a member of the Departmental Committee on the Dentists Act (1878), and his words, as set out in the succeeding paragraphs, serve to bring the importance of this matter yet more clearly home.

Health is Wealth.

As long ago as July 1917 the Lord President of the Council set up a Committee to inquire into the unsatisfactory state of the dental profession. The evidence tendered to that Committee was so conclusive that a unanimous report was issued and immediate legislation was recommended. Now that Parliament has reassembled, is it not possible for the Minister of Health to introduce an agreed measure which, in a few clauses, would translate the Committee's report into an Act of Parliament?

1. The closing of the profession to unregistered practitioners.
2. The admission to the Register of those who have carried on a *bona fide* practice for not less than five years.
3. The completion of an adequate system of school dental treatment, and the establishment of a public dental service.
4. The provision of full maintenance scholarships on a generous scale for dental mechanics and students wishing to qualify as dentists.

The Committee significantly remarked "Registered practitioners mainly attend to the dental needs of the upper and middle classes," and pointed out that as the artisan and working classes received very little treatment from the registered dentist, the unregistered man had supplied this necessary service.

At the present time there are 5,500 dental practitioners on the Register, and about 15,000 unregistered men, many of the latter being organised in one or two associations which have striven to maintain an ethical standard of practice.

It is neither fair to the public nor to these men who have served the workers that the present system should go on. There are grave evils attaching to the practice of dentistry by unqualified and uncontrolled persons, and widespread aggravation of sickness obtains by lack of attention to, or unskilful treatment of, the teeth. Indeed, some of the largest approved societies administering the National Insurance Act stated in evidence that "neglect of teeth trouble is the cause of quite half the ill-health found amongst the industrial classes, and of these a large majority of young women."

The extreme urgency of this matter, and the need for speedy legislation can best be stated in the words of the last paragraph of the Committee's report:

The dental profession should be regarded as one of the outposts of preventive medicine, and as such encouraged and assisted by the State. Treatment should be rendered available for all needing it. The present anomalous position in which an uneducated, untrained person can practise as a dentist, performing surgical operations on the teeth and jaws, doing untold damage and casting undeserved odium and dishonour on a scientific profession, is intolerable, and should be dealt with immediately.

